# Ectopic Eruption of First Permanent Molars in Children from North-Western Romania

**DOI:** 10.3390/diagnostics12112731

**Published:** 2022-11-08

**Authors:** Rahela Tabita Moca, Raluca Iulia Juncar, Abel Emanuel Moca, Denisa Tabita Sabău, Luminița Ligia Vaida, Mihai Juncar

**Affiliations:** 1 Doctoral School of Biomedical Sciences, University of Oradea, 1 Universității Street, 410087 Oradea, Romania; 2 Department of Dentistry, Faculty of Medicine and Pharmacy, University of Oradea, 10 Piața 1 Decembrie Street, 410073 Oradea, Romania

**Keywords:** ectopic eruption, first permanent molars, pediatric dentistry, orthodontics

## Abstract

Ectopic eruption of first permanent molars can lead to complications if left untreated. The aim of this study was to determine the prevalence of the ectopic eruption of first permanent molars in a sample of children from North-Western Romania, and to identify the characteristic of this anomaly in the studied population. We included patients aged between 5 and 9 years, and patients who needed a radiological examination for the diagnosis and treatment of dental or dento-maxillary diseases. The following exclusion criteria were applied: unclear or poor-quality radiographs; radiographs that belonged to patients who benefited from an orthodontic treatment before the panoramic radiograph was taken; patients who were undergoing orthodontic treatment when the radiograph was taken; and patients with local or general diseases that could influence dento-facial growth and development. Three degrees of severity were selected (moderate, severe, and very severe). The sample consisted of 438 patients, and 61 patients were diagnosed with ectopic eruption of first permanent molars (13.92%). Out of the 1752 analyzed molars, 103 were affected (5.87%). Patients with a moderate degree of ectopy were more frequently boys (56%, *n* = 14), while patients with a severe degree of ectopy were more frequently girls (52.8%, *n* = 19). Patients with a moderate degree of ectopy had significantly more frequently a unilateral position (57.1%, *n* = 16), while patients with a very severe degree of ectopy had significantly more frequently a bilateral position (36.4%, *n* = 12). The ectopic eruption was diagnosed at the level of the upper-right first permanent molar in a percentage of 18.4% (*n* = 19), at the level of the upper-left first permanent molar in a percentage of 17.5% (*n* = 18), at the level of the lower-right first permanent molar in a percentage of 32% (*n* = 33), and at the level of the lower-left first permanent molar in a percentage of 32% (*n* = 33). Although not very frequent, the ectopic eruption of first permanent molars is an important anomaly that should be early diagnosed, monitored and treated.

## 1. Introduction

Dental eruption is a physiological process at the end of which the erupting tooth will occupy its functional place on the dental arch and occlude with the opposing tooth. The process of tooth eruption can be negatively influenced by a variety of factors [1]. One of the issues that can arise in the process of dental eruption is ectopic eruption, a pathological situation in which the tooth does not follow its normal eruption path [2]. Among the teeth most frequently affected by this anomaly are the maxillary first permanent molars and maxillary canines, but the ectopic eruption can affect other teeth as well [3].

The first permanent molars erupt around the age of 6–7, distal to the second deciduous molars [4]. The ectopic eruption of the first permanent molars represents a local eruption anomaly that causes the first permanent molars to remain blocked under the distal surface of the second deciduous molar, leading to its inability to erupt up to the level of the occlusal plane and to the pathological resorption of the second deciduous molar’s roots [5]. The etiology of the ectopic eruption of first permanent molars is multifactorial, including local, genetic and inherited factors, and its prevalence is different in various populations worldwide [4,6]. Generally, the differences in prevalence are related to the size of the sample on which the study was carried out, the age range of the patients included in the study, or the dental status of the investigated patients [5].

The early diagnosis of ectopic eruption is necessary in order to prevent the development of other malocclusions and can be conducted with the help of routine panoramic radiographs [7]. The radiographic sign that indicates a possible ectopic eruption of the first permanent molars is the superior and mesial position of the first permanent molars [8]. Ectopic eruption can be classified into reversible and irreversible, the reversible form being the most common [9]. The treatment options for this anomaly depend on the patient’s age, the status of the second deciduous molar, the presence of the second premolar, and the severity of the ectopy, and include interproximal wedging and distal tipping of the first permanent molar [10]. The main goal of the early treatment of this pathology is to distance the ectopically erupting tooth from the deciduous tooth, to allow the normal eruption of the permanent tooth and to retain the deciduous molar on the dental arch until the physiological age of replacement [11].

The aim of this study was to analyze the prevalence and characteristics of the ectopic eruption of maxillary and mandibular first permanent molars in a sample of children from North-Western Romania.

## 2. Materials and Methods

### 2.1. Ethical Considerations

The study was conducted in accordance with the principles stated in the Declaration of Helsinki from 2008 and its later amendments. The research received the approval of the Ethics Committee of the Faculty of Medicine and Pharmacy, within the University of Oradea (IRB No. CEFMF/02 from 30 September 2022).

### 2.2. Sample Selection

This study was designed as a retrospective study and was based on the analysis of digital panoramic radiographs of a group of children from Oradea, North-Western Romania. The panoramic radiographs were collected from a private dental clinic in Oradea and were made with the Soredex Cranex Novus Panorex device (Soredex, Milwaukee, WI, USA).

All the panoramic radiographs were considered as necessary examinations and were required for a proper pedodontic or orthodontic treatment. After the initial clinical examination, the patients were given a recommendation for the panoramic radiography. The radiographs were dated and contained the name and date of birth of the patients. They were saved as a Joint Photographic Expert Group (JPEG) image. All parents or legal guardians signed an informed consent form by which they gave their permission that these radiographs could be used in the future for research purposes. The selected patients had a panoramic radiograph performed between 1 May 2020 and 31 August 2022.

Patients who met the following inclusion criteria were initially included in the study: patients aged between 5 and 9 years; patients who needed a radiological examination (panoramic radiography) for the diagnosis and treatment of dental or dento-maxillary diseases.

The following exclusion criteria were applied: unclear or poor-quality radiographs; radiographs that belonged to patients who benefited from an orthodontic treatment before the panoramic radiograph was taken; patients who were undergoing orthodontic treatment when the radiograph was taken; and patients with local or general diseases that could influence dento-facial growth and development.

### 2.3. Radiological Examination

**Figure 1:**
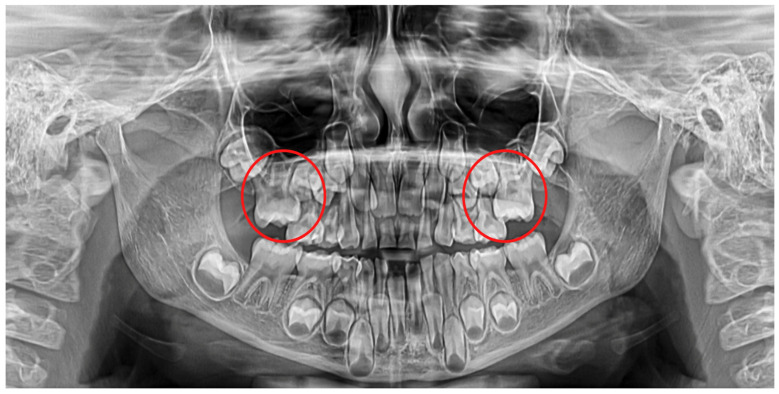
Ectopic eruption of upper first permanent molars.

**Figure 2:**
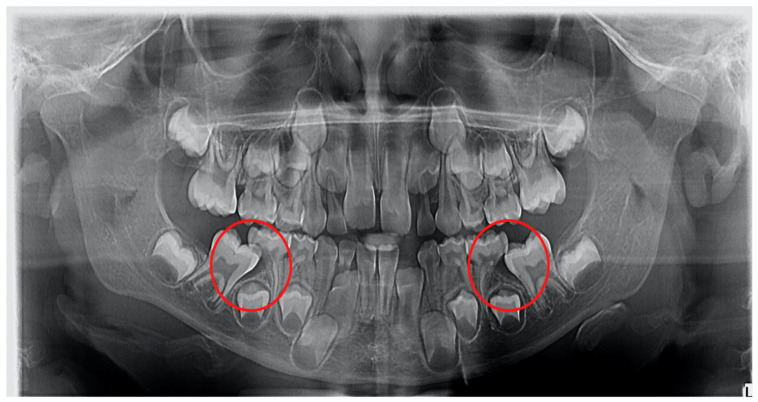
Ectopic eruption of lower first permanent molars.

The ectopic eruption of first permanent molars was classified according to the method proposed by Barberia Leache et al. (2005) [3]. This implies the existence of 4 degrees of severity, established according to the degree of pathological resorption of the second deciduous molar, caused by the ectopic eruption of the erupting first permanent molar. Thus, the severity can be mild (grade I), moderate (grade II), severe (grade III) and very severe (grade IV). However, due to the errors that can appear in the differentiation on the panoramic radiograph between the mild and moderate form (grade I and II), we classified the ectopic eruption according to only 3 degrees of severity (moderate, severe and very severe). The location of the affected first molars was also monitored. We investigated whether or not the ectopic molars were situated unilateral or bilateral, on the upper or on the lower dental arch, and on the right or on the left dental hemiarches. The type of affected molars was also investigated (Figure 1 and Figure 2).

The panoramic radiographs were first examined by one investigator (R.T.M.) and were double checked by another (A.E.M.). The inter-rater reliability was 96%, which showed a very good inter-rater agreement for the diagnosis of ectopic eruption.

### 2.4. Statistical Analysis

The statistical analysis was performed using IBM SPSS Statistics 25 (IBM, Chicago, IL, USA) and Microsoft Office Excel/Word 2021 (Microsoft, Redmond, DC, USA). Quantitative variables were tested for distribution using the Shapiro–Wilk test and were expressed as means with standard deviations or medians with interpercentile ranges. Quantitative independent variables with non-parametric distribution were tested using the Mann–Whitney U/Kruskal–Wallis H test.

Qualitative variables were expressed in absolute form or as a percentage. Differences between independent qualitative variables were tested using Fisher’s exact tests. Z-tests with Bonferroni correction were used to detail the results obtained in the contingency tables.

Power calculation and sample size estimation were made using GPower 3.1.9.7. We calculated the sample size for a contingency table using an effect size of 0.5, minimum power of 0.8, an alpha of 0.05 and the number of degrees of freedom equal to 2. A minimum number of 330 panoramic radiographs were required for this research.

## 3. Results

### 3.1. Socio-Demographic Characteristics

From the initial sample of 438 patients, 61 patients were diagnosed with ectopy of first permanent molars, representing a prevalence of 13.92%. 36 of the patients diagnosed with ectopic first permanent molars were girls (59%) and 25 were boys (41%).

The patients diagnosed with ectopic eruption of the first permanent molars were aged between 6–6.9 years (1.64%, *n* = 1), 7–7.9 years (19.67%, *n* = 12), 8–8.9 years (42.62%, *n* = 26) and 9–9.9 years (36.07%, *n* = 22). The average age of patients diagnosed with ectopy of first permanent molars was 8.13 ± 0.78 years (the median age being 8 years; IQR = 8–9 years). The age distribution in the group of girls and boys was non-parametric according to the Shapiro–Wilk test (*p* < 0.05), and the age differences between genders were insignificant according to the Mann–Whitney U test (*p* = 0.110); so, in this study, no significantly different age values were observed between boys and girls.

It was observed that from the initial sample of 1752 first permanent molars analyzed (belonging to 438 patients), 103 molars were diagnosed with ectopic eruption, and this represents a prevalence of 5.87% according to the total number of first permanent molars analyzed. The ectopic eruption was diagnosed at the level of the upper-right first permanent molar in a percentage of 18.4% (*n* = 19), at the level of the upper-left first permanent molar in a percentage of 17.5% (*n* = 18), at the level of the lower-right first permanent molar in a percentage of 32% (*n* = 33), and at the level of the lower-left first permanent molar in a percentage of 32% (*n* = 33). The location of the ectopic molars was relatively homogeneous, being slightly more frequent in the right hemiarches (50.5%, *n* = 52).

### 3.2. The Influence of Gender on the Ectopic Eruption of First Permanent Molars

The data in Table 1 represent the distribution of the patients according to gender and severity/location of the ectopic molars. Differences between groups were significant according to the Fisher test (*p* = 0.004), and Z tests with Bonferroni correction showed that patients with a moderate degree of ectopy were more frequently boys (56%, *n* = 14), while patients with a severe degree of ectopy were more frequently girls (52.8%, *n* = 19).

Regarding the distribution of patients related to gender and the location of the ectopy, it was observed that the differences between the groups were not significant according to the Fisher test (*p* = 0.296). However, unilateral ectopy was more frequent in girls (52.8%, *n* = 19), and bilateral ectopy was more frequent in boys (64%, *n* = 16).

The data in Table 2 represent the distribution of ectopic first permanent molars diagnosed according to gender and type of affected molar/affected dental arch/affected dental hemiarches. In girls, 3.6 (lower-left first permanent molar) was the most affected (29.8%, *n* = 17), and in boys, the most affected was 4.6 (lower-right first permanent molar) (39.1%, *n* = 18). However, the differences between the groups were not significant according to Fisher’s test (*p* = 0.239).

No significant differences were observed in terms of the distribution of ectopic first permanent molars in relation to gender and location on the dental arches and on the dental hemiarches, so gender did not influence the location of the affected molars.

### 3.3. The Severity of the Ectopic Eruption of First Permanent Molars

Out of 103 ectopic molars, 32 (33%) were moderately affected, 39 (34%) severely affected, and 32 (33%) very severely affected.

Regarding the distribution of patients according to the position and severity of ectopy, the tests showed that the differences between the groups were significant according to the Fisher test (*p* = 0.016), and Z tests with Bonferroni correction showed that patients with a moderate degree of ectopy were more frequently unilaterally affected (57.1%, *n* = 16), while patients with a very severe degree of ectopy were more frequently bilaterally affected (36.4%, *n* = 12) (Table 3).

The data in Table 4 represent the distribution of ectopic first permanent molars related to the degree of severity of ectopy and the type of affected molar/affected dental arch/affected dental hemiarch. Tooth 1.6 was severely affected in 23.1% of cases (*n* = 9), tooth 2.6 was severely affected in 20.6% of cases (*n* = 8), tooth 3.6 was moderately affected in 37.5% of cases (*n* = 12), and tooth 4.6 was very severely affected in 34.4% of cases (*n* = 11). The differences between the groups were not significant according to the Fisher test (*p* = 0.930).

The molars in the upper arch were severely affected in 43.6% of cases (*n* = 17), and those in the lower arch were very severely affected in 68.7% of cases (*n* = 22). Differences between groups were not significant according to the Fisher test (*p* = 0.468).

Regarding the distribution of ectopic molars related to the degree of severity of the ectopy and the location on the hemiarches, the tests showed that the differences between the groups were not significant (*p* = 0.854); so, in this study, no significant association was observed between the degree of severity of ectopy and location on the dental hemiarches.

## 4. Discussion

To the best of our knowledge, this study is the first in Romania to investigate the prevalence of ectopic eruption of first permanent molars, as well as the characteristics of this pathology in a sample of children from Romania. The etiology of the ectopic eruption of first permanent molars is complex. Macrodontia of the permanent teeth, macrodontia of the first molars, macrodontia of the second deciduous molars, a short and hypoplastic upper jaw, a delay in the calcification of the first permanent molars, or an abnormal eruption angle of the first permanent molars were incriminated as etiological factors [12]. If left untreated, the ectopic eruption of first permanent molars can lead to mesial angulation, loss of arch space, dental inclusion and ankylosis [13]. It can be considered a risk factor for maxillary constriction and severe crowding [12]. Early diagnosis is, therefore, essential in order to prevent the onset and development of complications [14]. Panoramic radiography is a necessary tool for an early diagnosis of this pathology [15]. Panoramic radiographs are frequently used in dentistry for establishing a diagnosis, but also as a screening tool for oral pathologies [16,17,18]. Anomalies such as hypodontia, hyperdontia, or impacted teeth can be diagnosed on panoramic radiographs [19]. In this study, consistent with the literature, the ectopic eruption of first permanent molars was diagnosed on panoramic radiographs.

In this study, the prevalence of ectopic eruption of first permanent molars was of 13.92% out of 438 patients. However, according to the number of investigated molars, from the total of 1752 first permanent molars, 5.87% of the cases were diagnosed with ectopic eruption. The prevalence obtained in this study is, generally, higher than in other studies identified in the literature. Hali et al. (2021) obtained a prevalence of 10.2%, higher than other similar studies, but still lower than in the present study [20]. Recent studies have been identified that reported a prevalence as low as 0.7% [21], and 2.2% [7]. A study carried out on Spanish children, with a sample size similar to the present study, reported a prevalence of 6.7%, half of that found in this research, but the authors only analyzed the maxillary first permanent molars [22]. High prevalence of ectopic first permanent molars was identified by da Silva Dalben et al. (2006) in a group of patients diagnosed with Treacher Collins syndrome. The authors reported a prevalence of 13.3%, which is similar to the present study [23].

The high prevalence obtained in this research can be explained by the fact that the panoramic radiographs belonged to patients who were examined with the purpose of initiating a pedodontic or orthodontic treatment. The differences in prevalence may be due to the genetic and ethnic populational differences [7], but also due to the different sample sizes, because a small sample increases the uncertainty of the prevalence [24].

Regarding the degree of severity of the ectopic eruption, the classification was made according to Barberia Leache et al. (2005), who used four degrees of severity, from mild to very severe [3]. However, it can be difficult to differentiate between mild and moderate forms of ectopy of first permanent molars on panoramic radiographs; thus, only three degrees of severity were used, these being moderate, severe and very severe. The classification with three degrees of severity was also used in a study from 2018, conducted on a population of children from Turkey [5]. The upper first permanent molars were severely affected in 43.6% of the cases, and 68.7% of the lower ectopic molars were very severely affected. Other studies have identified severe and very severe forms of ectopy more frequently in the upper arch [3,12]. Lower molars were more frequently affected, similar to other studies [5,25]. However, most studies show a higher frequency of this pathology in the upper arch [26,27].

No significant differences were observed between the right and left dental hemiarches. These results are similar and consistent with other studies identified in the literature [5,20,28]. Regarding the gender distribution, girls were more frequently affected than boys. However, generally, studies have reported a higher prevalence in boys [7,15].

The high prevalence in the studied population emphasizes the need for a better assessment of the eruption process of permanent teeth and especially of first permanent molars. The potential anomalies that can develop if this pathology is left untreated represent the main reason for the necessity of an early diagnosis.

Despite the fact that it is a novel study in the Romanian population, this research has some limitations. The sample size was limited, so a larger sample could lead to other results of the prevalence and of the characteristics of this pathology in Romanian children. We used the panoramic radiographs belonging to patients from a dental clinic located in Oradea, Romania, so most of the patients were from the North-Western region of Romania. A multicentric approach would be useful for obtaining a more accurate prevalence at a national level. The investigation of children from the general population, in addition to children requiring pedodontic or orthodontic treatment, would also be useful.

## 5. Conclusions

In this study sample, the ectopic eruption of first permanent molars had a prevalence of 13.92%, with girls being more affected than boys. Patients with a moderate degree of ectopy had a unilateral position significantly more frequently, while patients with a very severe degree of ectopy significantly more frequently had a bilateral position. The lower dental arch was more affected than the upper arch, but no significant differences were identified between the left and right dental hemiarches.

## Figures and Tables

**Table 1:** Distribution of the patients according to gender and severity/ location of the ectopic molars.

		Girls		Boys		*p* *
		No.	%	No.	%	
**Severity**	Moderate	10	27.8%	14	56%	0.004
	Severe	19	52.8%	3	12%	
	Very severe	7	19.4%	8	32%	
**Location**	Unilateral	19	52.8%	9	36%	0.296
	Bilateral	17	47.2%	16	64%	

* Fisher’s Exact Test.

**Table 2:** Distribution of the ectopic molars according to gender and type of molars/affected dental arch/affected dental hemiarch.

		Girls		Boys		*p* *
		No.	%	No.	%	
**Ectopic Molar**	1.6	14	24.6%	5	10.9%	0.239
	2.6	11	19.3%	7	15.2%	
	3.6	17	29.8%	16	34.8%	
	4.6	15	26.3%	18	39.1%	
**Dental Arch**	Upper	25	43.9%	12	26.1%	0.067
	Lower	32	56.1%	34	73.9%	
**Dental Hemiarch**	Right	29	50.9%	23	50%	1.000
	Left	28	49.1%	23	50%	

* Fisher’s Exact Test.

**Table 3:** Distribution of the patients according to the position of the affected molars and the severity of ectopic eruption.

		Unilateral		Bilateral		*p* *
		No.	%	No.	%	
**Severity**	Moderate	16	57.1%	8	24.2%	0.016
	Severe	9	32.1%	13	39.4%	
	Very severe	3	10.7%	12	36.4%	

* Fisher’s Exact Test.

**Table 4:** Distribution of the ectopic molars according to severity and type of molar/affected dental arch/affected dental hemiarch.

		Moderate		Severe		Very Severe		*p* *
		No.	%	No.	%	No.	%	
**Ectopic Molar**	1.6	5	15.6%	9	23.1%	5	15.6%	0.930
	2.6	5	15.6%	8	20.5%	5	15.6%	
	3.6	12	37.5%	10	25.6%	11	34.4%	
	4.6	10	31.3%	12	30.8%	11	34.4%	
**Dental Arch**	Upper	10	31.3%	17	43.6%	10	31.3%	0.468
	Lower	22	68.7%	22	56.4%	22	68.7%	
**Dental Hemiarch**	Right	15	46.9%	21	53.8%	16	50%	0.854
	Left	17	53.1%	18	46.2%	16	50%	

* Fisher’s Exact Test.

## Data Availability

The data presented in this study are available on request from the corresponding authors. The data are not publicly available due to privacy reasons.
